# Outcome measures for ambulant children with cerebral palsy after limb extremity orthopedic surgery: What should be measured and who should decide?

**DOI:** 10.1111/dmcn.70181

**Published:** 2026-02-11

**Authors:** Elizabeth R. Boyer

**Affiliations:** ^1^ Gillette Children's Specialty Healthcare – Research Saint Paul MN USA; ^2^ Rehabilitation Science University of Minnesota‐Twin Cities Minneapolis MN USA; ^3^ Orthopedic Surgery University of Minnesota Twin‐Cities Minneapolis MN USA

## Abstract

**This commentary is on the original article by Almoajil et al. on pages** 1127‐1138 **of this issue.**

Currently, there is no established expectation or enforcement of standard outcome measures in pediatric cerebral palsy (CP) intervention studies, making it difficult to confidently translate research findings into the clinical setting, where comparison of treatment effects and reasonable alternatives should be measured on a common scale. The paper by Almoajil et al.^1^ is a high‐impact study that completes the group's journey to identify orthopedic surgery outcomes for children with CP. Like all Core Outcome Measures in Effectiveness Trials (COMET) core outcome set (COS) studies, its goal is to improve comparability across studies, a noble and necessary aspiration. A related effort was undertaken with common data elements.^2^ Patients/parents, clinicians, and researchers all want to know how likely, and to what extent, a given intervention (vs another intervention or no intervention) will help specific outcomes. A COS helps answer this question and support shared decision‐making.

Key study strengths were the methodological rigor, consideration of real‐world and widespread feasibility, and in the early phases, international representation and engagement of people with lived experience (PWLE).^3^ This study is one of 11 COS studies for CP on the COMET Initiative's website. There is an added benefit when COS measures agree across interventions (e.g. Gross Motor Function Measure, instrumented gait analysis, CP‐Quality of Life for Children questionnaire), as this enables additional comparison of treatment efficacy.

I am curious how recommended outcome measures would have differed if PWLE were included in this final phase, or if the authors had not constrained themselves to existing outcomes in the specialized field of elective pediatric CP lower extremity orthopedic surgery. Partnering with PWLE to conduct research has many synonyms (e.g. community‐engaged research, participatory action research, community‐based participatory research), but the ideal state is the same—one where PWLE are involved as equal partners from start to finish.^4^ PWLE offer novel and valuable perspectives. Had PWLE been consulted after the literature review, they may have pointed out aspects of fatigue, pain, balance, walking, self‐esteem, or independence (e.g. caregiver burden) that are not fully captured by the recommended outcome measures. Restricting candidate outcomes to those historically used in orthopedics can be suboptimal, since outcomes were likely chosen by clinicians or researchers without input from PWLE and it disregards quality research from other fields. The GOAL^TM^ (Gait Outcomes Assessment List), recommended across all eight COS domains,^1^ partly mitigates this concern since it was developed with strong patient/parent input. Like all patient‐reported outcomes, responses may be influenced by perceptions, social pressures, and other biases. This could partly explain the weak to moderate agreement between GOAL and lab‐based measures.^5^ Still, performance‐based tests have limitations (e.g. variable patient effort, responsiveness), so both types of measures are important.^1^

Balance and falls were important outcomes,^1^, ^3^ consistent with our findings,^5^ yet they are rarely measured in this population. Fall incidence and performance‐based tests like the Balance Evaluation Systems Test (BESTest) should be considered since it measures reactive balance, unlike the Pediatric Balance Scale. Reactive balance is important due to its relation to fall recovery. Future clinical trials may consider patient‐centered outcomes outside the seven core measures recommended,^1^ reinforcing a key point that COS are meant to be the minimal set.

The main goal of developing a COS is comparison, namely across studies, but comparison across time is also valuable. Many adults with CP report increased fatigue, pain, balance problems, and functional decline with age. Ideally, a COS uses measurements that are valid across the lifespan to allow us to understand if or how pediatric orthopedic surgery alters these trajectories. This should be explored further.

My closing challenge to the field is implementation. A combined top‐down and bottom‐up approach is needed, involving professional organizations, funders, industry, collaborative networks (e.g. CP Research Network, CP Global Clinical Trials Network), clinical trialists, and investigators. Broad adoption of this COS or related common data elements will improve efficiency, transparency, and our ability to answer patients' primary question: which treatment is right for me?

## Data Availability

Not required.
